# Automated Detection of COVID-19 Using Machine Learning Analysis of White Blood Cell Flow Cytometry Images

**DOI:** 10.7759/cureus.107140

**Published:** 2026-04-16

**Authors:** Akihiro E Hirosse, André R Backes, Renata S Woloszynek, Fabio F Neves

**Affiliations:** 1 Department of Medicine, Federal University of São Carlos, São Carlos, BRA; 2 Department of Computing, Federal University of São Carlos, São Carlos, BRA; 3 Department of Laboratory Medicine, University Hospital Federal University of São Carlos, São Carlos, BRA

**Keywords:** artificial intelligence, cell morphological data, cell population data, covid-19, flow cytometry, hemogram, image processing, machine learning, sars-cov-2

## Abstract

Background: COVID-19 is usually diagnosed by real-time polymerase chain reaction testing of nasopharyngeal swabs, but this approach may be associated with a prolonged time to diagnosis. Machine learning incorporating findings from standard laboratory tests may more rapidly identify individuals infected with SARS-CoV-2, enabling early management and protection for healthcare staff.

Methods: A machine learning model was developed to detect patients infected with COVID-19 using only white blood cell flow cytometry without using symptom or clinical history data. The model included images from 106 patients positive for SARS-CoV-2 and 211 controls admitted to a university hospital with respiratory symptoms. A total of 17 texture-feature analysis methods were tested using three classifiers. Finally, the Particle Swarm Optimization algorithm was used to combine the best-performing models. The performance of the final model was assessed using the area under the receiver operating characteristic curve (AUROC), sensitivity, specificity, precision, and F1-Score.

Results: Using the five-fold cross-validation technique, the final model achieved an accuracy of 88.96% for diagnosing patients with a SARS-CoV-2 infection. It also had a sensitivity of 78.30%, a specificity of 94.31%, a precision of 87.83%, an F1-score of 0.83, and an AUROC of 0.86.

Conclusions: The final algorithm showed good diagnostic performance even when compared with models that included clinical, epidemiological, and other laboratory or ancillary test data. The use of cytometry images could represent a significant advance in the early diagnosis of COVID-19.

## Introduction

Severe acute respiratory syndrome coronavirus 2 (SARS-CoV-2) is most infectious in the early stages. Therefore, prompt screening of symptomatic individuals is essential to determine who must be isolated and tested. However, symptom-based screening detects only approximately half of transmitters, because asymptomatic individuals or those with only mild symptoms also transmit the virus [1]. This pattern of absent or mild symptoms is highly prevalent, as observed in a meta-analysis that evaluated the symptoms of 24,410 adult patients with coronavirus disease 2019 (COVID-19) and found that 22% did not have a history of fever and 43% denied having a cough [2]. Importantly, the diagnostic yield of symptom-based screening may vary according to viral variant, vaccination status, timing of clinical assessment, disease severity, and study setting, which should be considered when interpreting the generalizability of these estimates.

Transmission of SARS-CoV-2 in healthcare settings has contributed significantly to the global burden of COVID-19 because healthcare settings are particularly vulnerable to the spread of the virus owing to the dense concentrations of susceptible patients, elevated frequencies of risky medical procedures, and high rates of interpersonal contact [3]. A study in the Stockholm region reported an incidence of 1.57 nosocomial SARS-CoV-2 infections per 1,000 patient-days and an excess mortality risk associated with the infection [4].

The pathophysiology of SARS-CoV-2 infection involves dysregulation of the innate immune system, which is responsible for initial antiviral activity essential for the host response and illness protection [5]. The monocyte population is intimately involved as a trigger and target of the innate cellular immune response in patients with COVID-19, and the analysis of this population of cells can assume both diagnostic and prognostic value. A systematic review and meta-analysis published in 2023 concluded that monocyte distribution width has clinical value in diagnosing and stratifying disease severity and can predict the need for mechanical ventilation and death [6].

Studies have shown that computer-aided diagnostic systems enable accurate diagnosis of various diseases from medical images and data, speeding up the process of analysis, thus enabling an early start to treatment. One method to achieve this is through the use of machine learning, a branch of artificial intelligence (AI) that uses algorithms to enable AI to imitate human learning, gradually improving its accuracy. Machine learning algorithms use different data types, such as categorical, numeric, free text, images, or sounds.

Some studies have used machine learning models to diagnose SARS-CoV-2 infection from numeric hemogram parameters, obtaining an accuracy of approximately 90% [7,8]. Due to the high prevalence of atypical monocytes with large coalescing cytoplasmic vacuoles, in patients with a SARS-CoV-2 infection [8], we hypothesized that the analysis of white blood cell flow cytometry images using machine learning models would achieve greater diagnostic accuracy compared to the analysis of numerical parameters. Thus, we aimed to develop a machine learning model using white blood cell cytometry images, which is efficient in identifying patients with respiratory symptoms and a SARS-CoV-2 infection.

## Materials and methods

Study design and participants

This diagnostic accuracy study was performed using two datasets of de-identified laboratory records of adult patients hospitalized with suspected COVID-19 from July 2022 to March 2024 at the University Hospital in São Carlos, Brazil. The first dataset was the flow cytometry images obtained from an ADVIA 560 Hematology System (Siemens Healthcare Diagnostics Inc., Tarrytown, NY, USA), and the second was the results of real-time polymerase chain reaction (RT-PCR) assays for SARS-CoV-2 in patients hospitalized due to respiratory symptoms. Thus, all included individuals were assessed in an in-hospital symptomatic setting, and the laboratory images reflected the hematological profile at the time of hospital evaluation rather than a standardized stage of infection. SARS-CoV-2 was detected using the TaqMan 2019-nCoV Assay Kit v1 (Thermo Fisher Scientific Inc., Waltham, MA, USA), which was designed to detect SARS-CoV-2-specific RNA. Molecular methods such as RT-PCR have excellent sensitivity and specificity for detecting SARS-CoV-2 [9].

A total of 106 patients positive for SARS-CoV-2 and 211 controls (negative RT-PCR) were selected for modeling the machine learning algorithms. The investigators had access only to laboratory data, including SARS-CoV-2 RT-PCR results and hematology analyzer images, and did not have access to complete clinical or microbiological records for RT-PCR-negative patients. Therefore, it was not possible to determine the specific etiologies of respiratory illness in the control group. No clinical or demographic features were used in the AI model. The study protocol complied with the principles of the Declaration of Helsinki and was approved by the Federal University of São Carlos Ethical Board (approval number 7838.5424.6.0000.5504).

Texture analysis

**Figure 1:**
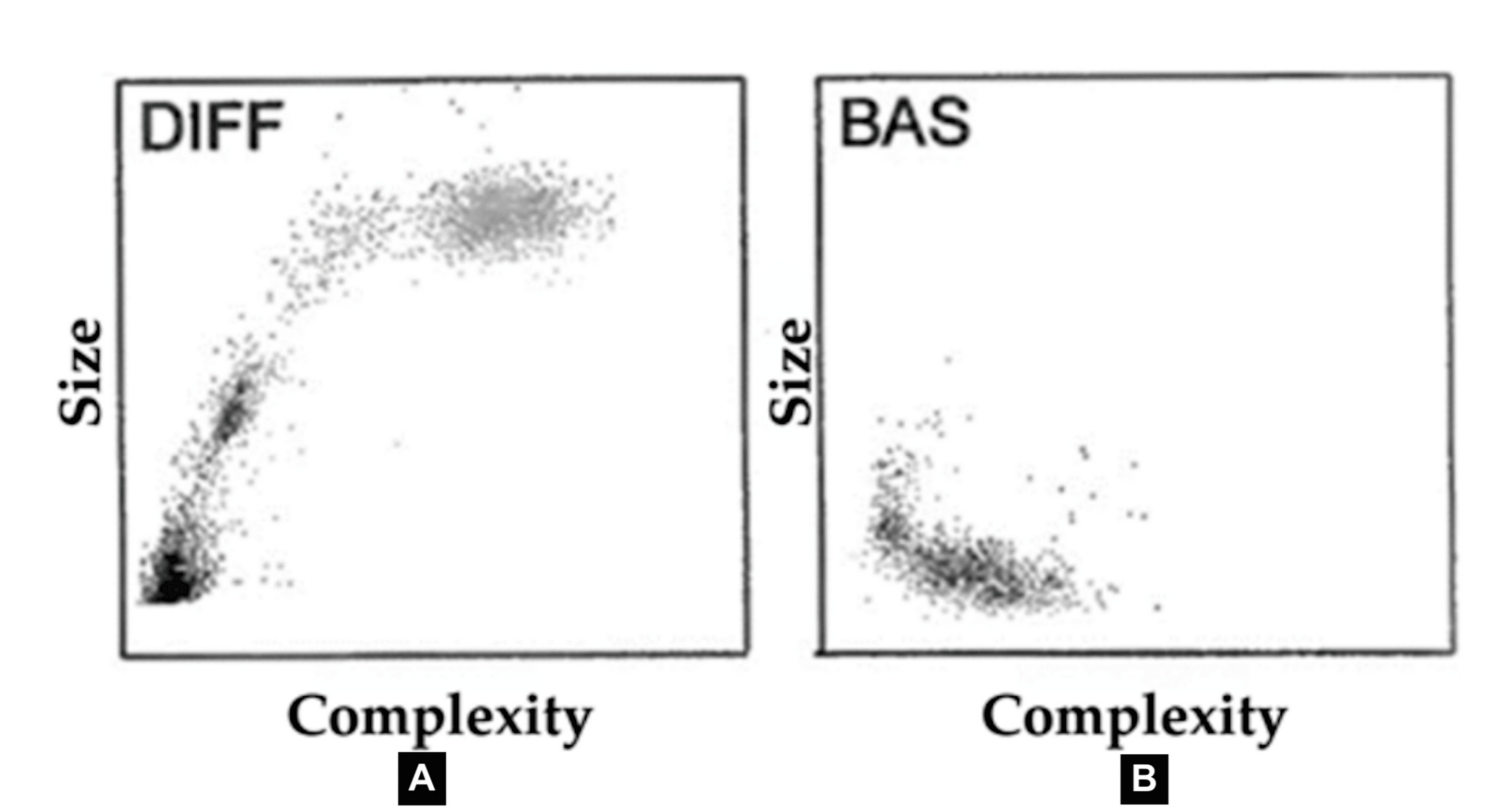
Representative white blood cell flow cytometry images used for model development. (A) White blood cell differential (DIFF) image; (B) basophil image. Both were obtained from the hematology analyzer. These images were digitized from routine complete blood count reports and used as input for texture feature extraction and machine learning classification.

Images were digitized from printed complete blood cell count reports, and those related to the white blood cell differential (DIFF) and basophils (BAS) were analyzed (Figure 1). Image edges were removed to avoid bias in the AI analysis. To minimize variability and artifacts, we adopted a standardized acquisition and preprocessing pipeline. All images were digitized under consistent conditions, with fixed resolution and controlled scanning parameters. Preprocessing steps were applied uniformly across the dataset, including resizing to a common spatial resolution and, when necessary, intensity normalization and contrast adjustment. In addition, region-of-interest selection followed a simple but consistent protocol, as they were always located at the same region of the image.

Texture features analysis methods based on results reported in the literature and their wide range of applications and novelty were selected. Each method is described in Table 1.

**Table 1:** Selected texture features analysis methods Source: Created by the authors.

Name	Description
Fourier descriptors	This method uses the two-dimensional Fourier transform and a shifting operator applied to the resulting spectrum to compute spectral descriptors [10]. In this study, 259 descriptors were calculated, each representing the sum of all absolute coefficients at the same radial distance from the center of the image.
Gabor filter	A family of filters used in image processing to extract features from images at various scales and orientations. It was inspired by the receptive fields of simple cells in the visual cortex and is particularly effective at capturing local features, such as edges, textures, and orientations [11]. Twenty-four filters were computed (six rotation filters and four scale filters) with lower and upper frequencies of 0.05 and 0.4, respectively.
Wavelet descriptors	For a given texture image, the Daubechies 4 wavelet was applied to perform three levels of dyadic decomposition [12]. From each decomposition, the energy and entropy of the horizontal, vertical, and diagonal detail coefficients were calculated, resulting in 18 descriptors.
Haralick	This method performs texture description using the joint probability distributions between pairs of pixels at specific distances and directions [13]. Nonsymmetric matrices with angles θ = {0°, 45°, 90°, 135°} and distances d = {1, 2} were used. From each matrix, energy and entropy were computed, yielding a total of 16 descriptors.
Discrete Cosine Transform (DCT)	This method uses three 1D Discrete Cosine Transform (DCT) basis vectors (U1 = [1, 1, 1]ᵀ, U2 = [1, 0, −1]ᵀ, and U3 = [1, −2, 1]ᵀ) to generate eight 3 × 3 DCT masks [14]. Each mask was applied to the input texture image, and the local variance of the output was computed, resulting in a total of eight descriptors.
Gray Level Dependence Matrix (GLDM)	This method analyzes the frequency of occurrence between two pixels with a specified absolute intensity difference, considering a given distance and inter-sample space [15]. Four distances: (0, d), (−d, d), (d, 0), and (−d, −d) were used, together with three inter-sample spaces: 1, 2, and 5. From each combination, five measurements: contrast, angular second moment, entropy, mean, and inverse difference moment were computed, resulting in a total of 60 descriptors.
Local Binary Patterns (LBP)	This method describes texture by analyzing histograms of Local Binary Patterns (LBP), which capture the spatial configuration of local features within an image [16]. Three different configurations: (P, R) = {(8, 1), (16, 2), (24, 3)} were evaluated, resulting in three distinct histograms.
Fractal descriptors	The log-log curve derived from a fractal dimension method was used to characterize the complexity of the texture patterns. Three approaches for computing the log-log curve were evaluated: Bouligand-Minkowski, mass-radius, and mean square difference [17].
Lacunarity	This method quantifies the spatial dispersion of gaps of a specific size to describe a texture pattern [18]. The gliding-box approach was used together with two threshold methods—local and global—to compute the gaps.
Lacunarity 3D	This method treats gray level intensity from an image as a third dimension, applying the gliding-box approach to measure the number of gaps within a 3D grid of the image [18].
Differential lacunarity	This method assesses the local difference between the minimum and maximum gray levels using a gliding-box approach to quantify the number of gaps in a texture image [19].
Joint Adaptive Median Binary Patterns (JAMB)	A local texture descriptor that combines the strengths of both adaptive median filtering and LBP. It is designed to be robust to noise and illumination variations while providing discriminative information for texture classification. This technique results in a feature vector containing 320 descriptors representing the local microstructure of the image [20].
First-order statistics	Five descriptors were computed from the image histogram: mean, variance, kurtosis, energy, and entropy [21].

Machine learning algorithms

After image preprocessing, each basophil (BAS) and differential (DIFF) image was converted into a numerical feature vector by applying the selected texture analysis methods described above. These feature vectors were then used as input for supervised classification into the SARS-CoV-2-positive or control group. All image preprocessing steps, texture feature extraction procedures, classifier implementation, and performance calculations were carried out in MATLAB using in-house routines developed by the authors. For Support Vector Machine (SVM) classification, the LIBSVM library was used with default parameter settings. Brief descriptions of the classifiers are provided below.

K-Nearest Neighbors (KNN)

This classifier uses instance comparison and a voting mechanism to classify the samples [22]. The KNN algorithm compares each input with all the training samples using a similarity metric, selecting the KNN. The input is then assigned to the most common class among the K-nearest samples. In the experiments, the Euclidean distance was used as the similarity metric, with K set to 1, and the features were standardized for improved accuracy.

Linear Discriminant Analysis (LDA)

This statistical classifier is based on Fisher’s discriminant function. It relies on Bayes’ theorem and seeks to find a linear combination of features that best separates different classes [23]. To achieve this, LDA considers the mean feature vector (µi) for each class and assumes that the covariance matrix (Σ) of the features is equal across all classes.

Support Vector Machine (SVM)

This classifier is based on statistical learning frameworks designed to find a hyperplane in an N-dimensional space that optimally separates two sets of data points. The best hyperplane is defined as the one that maximizes the margin, which is the distance between the nearest data points in the two sets. In this study, LIBSVM was used with the default parameter settings [24].

The use of hyperparameters ensures a fair and unbiased comparison between methods and classifiers, without introducing additional computational cost and complexity that may obscure the interpretability and reproducibility of the results.

To validate these classifiers, five-fold cross-validation was used, reporting the mean and standard deviation of the accuracy across the folds. This allows the model to be trained and evaluated on multiple distinct splits of the data, thereby providing a more robust estimate of its generalization performance and reducing the probability of overfitting. This procedure was conducted at the patient level, since each sample (image) represents an independent patient instance. This prevents any overlap of patient-specific information between training and testing folds, thereby eliminating the risk of data leakage associated with having images from the same patient in both sets. In addition, we used stratified cross-validation, ensuring that the class distribution of the target variable was preserved across all folds, thus avoiding imbalanced splits and providing a more reliable and consistent evaluation.

Finally, to combine the best-performing texture models, the Particle Swarm Optimization (PSO) technique, an optimization algorithm inspired by the social behavior of bird flocks or fish shoals, was used [25]. We used PSO to identify the optimal combination of texture methods. We represented the combination of methods as a particle positioned at [X(1),X(2),…,X(D)], where D is the number of methods (i.e., search space dimensions). If X(i) ≥ 0.5, all descriptors of the method are selected to compose the feature vector evaluated. We used the accuracy of the selected combination as the fitness function.

Model performance measures

The accuracy of the performance of each preliminary model was determined. The sensitivity, specificity, area under the receiver operating characteristic curve (AUROC), and F1-score of the most accurate model were also determined. The F1 score is used in machine learning to evaluate the performance of the classification models. It combines two essential metrics: precision and recall. Precision is the proportion of positives that are genuinely positive, and recall is the proportion of positives that are correctly classified as positive.

## Results

Baseline characteristics of the patients

The COVID-19 group had a higher percentage of females and a higher mean ±standard deviation age (58% female, 62.7±28.5 years) than the control group (51% female, 48.4±34.0 years). All participants had respiratory symptoms, and COVID-19 was clinically suspected. Because the study included hospitalized adults, the findings should be interpreted within a symptomatic in-hospital population and may not directly reflect patients assessed earlier in the course of infection or in milder outpatient settings. Clinical and epidemiological data were not used for AI modeling.

Models based on individual images (BAS or DIFF)

For the isolated BAS images, the KNN method with the discrete cosine transform (DCT) statistical model (71.64%±3.07%), the LDA method with the global lacunarity model (73.86%±5.93%), and the SVM method with the GLDM model (75.74%±4.84%) were the most accurate.

For the isolated DIFF images, the KNN method in the Joint Adaptive Median Binary Patterns statistical model (72.54%±4.40%), the LDA method in the Global Lacunarity model (81.69%±4.65%), and the SVM method in the DCT model (80.74%±2.72%) were the most accurate.

Models based on combined images (BAS and DIFF)

When both samples were used in the analysis, a significant improvement in accuracy was observed in several of the statistical methods. The greatest improvements were the KNN model using the Gabor filter method, with 75.70% ± 4.74% accuracy; the LDA model using the Lacunarity 3D method (83.90% ± 5.32%); and the SVM model using the Fourier descriptors method (85.17% ± 6.22%), as shown in Table 2.

**Table 2:** Accuracy of each method using BAS plus DIFF images BAS: basophils; DIFF: white blood cell differential; KNN: K-nearest neighbors; LDA: linear discriminant analysis; SVM: support vector machine.

Method	KNN	LDA	SVM
Fourier descriptors	72.54 ± 2.95	80.41 ± 3.61	85.16 ± 6.22
Gabor filter	75.70 ± 4.74	72.24 ± 1.91	77.27 ± 1.89
Wavelet descriptors	67.85 ± 5.95	69.43 ± 3.76	74.46 ± 2.38
Haralick	64.34 ± 3.58	79.49 ± 3.37	79.48 ± 3.25
Discrete Cosine Transform	66.94 ± 5.86	78.21 ± 4.06	78.20 ± 3.05
Gray Level Dependence Matrix	70.35 ± 5.81	-	79.50 ± 1.82
Local Binary Pattern	69.14 ± 5.80	73.81 ± 1.77	78.53 ± 1.91
Fractal (Bouligand-Minkowski)	66.89 ± 4.45	77.93 ± 3.56	77.56 ± 5.08
Fractal (average square difference)	33.43 ± 0.22	-	66.56 ± 0.22
Fractal (Mass-Radius)	68.42 ± 4.52	72.21 ± 3.15	79.80 ± 5.19
Fractal error (inferior/superior)	72.54 ± 2.95	79.79 ± 4.00	82.00 ± 3.73
Lacunarity (global)	68.42 ± 6.01	83.88 ± 5.01	80.11 ± 3.34
Lacunarity (local)	70.34 ± 2.61	82.33 ± 3.39	81.05 ± 5.22
Lacunarity 3D	68.42 ± 6.01	83.90 ± 5.32	80.11 ± 3.34
Differential lacunarity	33.43 ± 0.22	-	66.56 ± 0.22
Joint Adaptive Median Binary Patterns	74.46 ± 4.12	-	80.12 ± 2.63
First order	33.43 ± 0.22	-	66.56 ± 0.22

Statistical analyses were performed independently, allowing results to be interposed. In this context, further testing was performed using the PSO technique, including all the statistical methods listed above.

The most significant result was obtained using the SVM model, which grouped the Fourier descriptors, Haralick, LBP, global lacunarity, 3D lacunarity, and fractal (mass-radius) statistical methods, obtaining a diagnostic accuracy of 88.96% (Table 3).

**Table 3:** Selected texture descriptors and final accuracy, using the Particle Swarm Optimization algorithm BAS: basophils; DIFF: white blood cell differential; KNN: K-nearest neighbors; LDA: linear discriminant analysis; SVM: support vector machine.

Method	KNN	LDA	SVM
Fourier descriptors	X	X	X
Gabor Filter	X		
Wavelet descriptors			
Haralick			X
Discrete Cosine Transform		X	
Gray Level Dependence Matrix			
Local Binary Pattern	X		X
Fractal (Bouligand-Minkowski)			
Fractal (Average square difference)			
Fractal (Mass-Radius)			X
Fractal error (inferior/superior)	X		
Lacunarity (global)			X
Lacunarity (local)	X		
Lacunarity 3D			X
Differential lacunarity		X	
Joint Adaptive Median Binary Patterns			
First order			
Success rate	76.95	85.18	88.96

**Figure 2:**
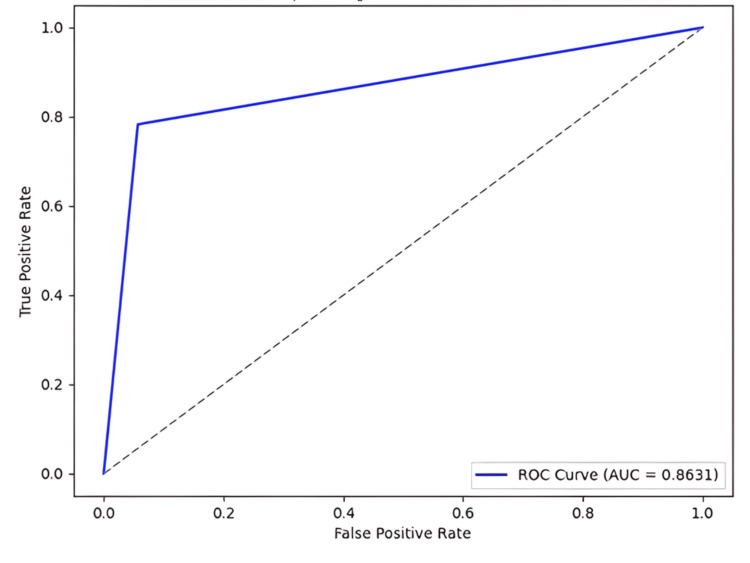
ROC curve of the final model, obtained using five-fold cross-validation. The final model corresponds to the Support Vector Machine (SVM) algorithm applied simultaneously to six texture descriptors (Fourier descriptors, Haralick, Local Binary Pattern, Fractal (Mass-Radius), Lacunarity (global), and Lacunarity 3D). The AUC is presented with its 95% confidence interval (0.750–0.976), estimated using DeLong’s method, and p-value < 0.001. AUC, area under the curve; ROC, receiver operating characteristic.

The final model also had a sensitivity of 78.30%, a specificity of 94.31%, a precision of 87.83%, an F1-score of 0.83, and an AUROC of 0.86 (Figure 2). The moderate sensitivity value reflects the inherent trade-off between sensitivity and specificity, which is influenced by the decision threshold used to convert predicted probabilities into class labels. In the current study, default decision thresholds were adopted to ensure a fair and standardized evaluation. Nevertheless, the model can be adjusted to favor higher sensitivity by tuning the decision threshold, for instance by lowering the classification cutoff or adopting class-specific thresholds in a one-vs-rest setting. Such adjustments would increase the true positive rate at the expense of a higher false positive rate, which may be acceptable in screening scenarios.

## Discussion

We developed a machine learning model to identify adult patients infected with SARS-CoV-2 using only cytometry images of white blood cells. Even without the use of sociodemographic or clinical features, the developed model showed excellent accuracy in predicting SARS-CoV-2 infection in symptomatic individuals. Since a complete blood cell count is frequently ordered for patients with infections, model-based testing for SARS-CoV-2 can be performed on a large scale, with a meager added cost. With this model, clinicians could be alerted promptly to patients at risk of COVID-19, enabling rapid quarantine measures for high-risk patients and prompting rapid retesting with a gold-standard examination.

A single-center study [26] that evaluated 5,893 patients in New York City proposed a machine learning model incorporating extensive laboratory blood tests and demographic features to predict SARS-CoV-2 infection. Despite the excellent accuracy observed (AUROC 0.85; 95% confidence interval (CI), 0.83-0.88), the weakness of this model lies in the difficulty of comparing subtle differences in laboratory test results between infected and non-infected individuals in the early stages of the disease, as well as the absence of all laboratory tests used in the model in a clinical setting. For this model to be widely used, it is necessary to standardize the COVID-19-like laboratory test panel, including all tests used for modeling.

A large multicenter study [27] was performed using a machine learning model developed from datasets of 1,041 clinical centers across the United States, including 64 routine laboratory tests in the model. After external validation, there was excellent diagnostic accuracy (AUROC 0.91; 95% CI, 0.90-0.92). Despite the high methodological quality of the study, the authors used controls from a different timeframe (pre-pandemic), which could introduce bias. Furthermore, the use of 64 laboratory parameters may limit the application of the model in low-resource settings.

Gómez-Rojas et al. [8] conducted a study similar to the present study using only numerical hemogram parameters. This decision was made because it is inexpensive to perform and can be completed in less than one hour; therefore, it could be available at all clinical centers. Blood cell count analysis can be a highly sensitive diagnostic method because there is evidence of early changes in the number and distribution of leukocytes [6]. Machine learning algorithms can incorporate multiple parameters from a hemogram, assessing individual cells simultaneously with a more rapid analysis. High diagnostic accuracy was observed using only white blood cell count parameters, with an AUROC of 0.95, sensitivity of 0.89, specificity of 0.90, a positive predictive value of 0.82, and a negative predictive value of 0.94.

In contrast to previous machine learning models, our model does not require input of clinical or laboratory data and can be integrated with the hematology analyzer, alerting staff to the possibility of SARS-CoV-2 infection, even when the diagnosis has not been considered. Our contribution to scientific knowledge is the development and validation of a machine learning model for diagnosing COVID-19 using only cytometry images.

From a biological perspective, the texture descriptors selected by the final model may capture subtle alterations in leukocyte morphology, spatial organization, and cytoplasmic heterogeneity associated with monocyte activation in COVID-19 [28], even though these descriptors do not map directly onto a single cellular structure. Rather than replacing morphological assessment, this computational approach may detect complex multiscale image patterns that are difficult to quantify consistently by routine visual inspection alone [29]. Accordingly, the potential clinical applicability of this approach should be interpreted as preliminary and dependent on successful external validation across other hospitals, patient populations, and laboratory platforms [30].

Finally, there are potential limitations to the use of this model. First, this was a single-center study with a small sample size. Second, the model was developed from an in-hospital cohort and may not be directly applicable to less complex settings. Third, all images were obtained from a specific hematology analyzer and digitized from printed reports, so instrument-related and digitization-related variability may have influenced model performance. Fourth, although cross-validation was conducted at the patient level to reduce the risk of data leakage, the absence of external validation means that some degree of optimistic performance estimation cannot be excluded. Fifth, individual SARS-CoV-2 variant typing was not available at our study site; however, regional and national genomic surveillance indicates that Omicron and its sub-lineages predominated during the study period. Finally, we did not stratify results according to vaccination status, symptom duration, or disease severity, which may also affect generalizability.

## Conclusions

This study demonstrates that machine learning analysis of white blood cell flow cytometry images can accurately identify patients with SARS-CoV-2 infection using only routinely generated laboratory images, without the need for clinical or demographic data. By leveraging information already produced during a standard complete blood count, this approach may enable rapid, automated screening for COVID-19 with minimal additional cost. If integrated into hematology analyzers, such systems could assist clinicians by flagging patients at risk of infection, facilitating earlier diagnostic confirmation, timely isolation measures, and prompt initiation of therapy. However, these findings should be interpreted cautiously, given the single-center design, the use of a specific analyzer, the lack of external validation, and the possibility that image digitization and residual sources of bias may have influenced performance estimates. Larger multicenter studies with independent external validation are required before broader clinical implementation.
